# The prevalence and factors associated with obesity among adult women in Selangor, Malaysia

**DOI:** 10.1186/1447-056X-8-2

**Published:** 2009-04-09

**Authors:** Sherina Mohd Sidik, Lekhraj Rampal

**Affiliations:** 1 Department of Community Health, Faculty of Medicine and Health Sciences, Universiti Putra Malaysia, Malaysia

## Abstract

**Introduction:**

The prevalence of obesity in developing countries especially among women is on the rise. This matter should be taken seriously because it can burden the health care systems and lower the quality of life.

**Aim:**

The purpose of this study was to determine the prevalence of obesity among adult women in Selangor and to determine factors associated with obesity among these women.

**Methods:**

This community based cross sectional study was conducted in Selangor in January 2004. Multi stage stratified proportionate to size sampling method was used. Women aged 20–59 years old were included in this study. Data was collected using a questionnaire-guided interview method. The questionnaire consisted of questions on socio-demographic (age, ethnicity, religion, education level, occupation, monthly income, marital status), Obstetric & Gynaecology history, body mass index (BMI), and the Patient Health Questionnaire (PHQ-9).

**Results:**

Out of 1032 women, 972 agreed to participate in this study, giving a response rate of 94.2%. The mean age was 37.91 ± 10.91. The prevalence of obesity among the respondents was 16.7% (mean = 1.83 ± 0.373). Obesity was found to be significantly associated with age (p = 0.013), ethnicity (p = 0.001), religion (p = 0.002), schooling (p = 0.020), educational level (p = 0.016), marital status (p = 0.001) and the history of suffering a miscarriage within the past 6 months (p = 0.023).

**Conclusion:**

The prevalence of obesity among adult women in this study was high. This problem needs to be emphasized as the prevalence of obesity keeps increasing, and will continue to worsen unless appropriate preventive measures are taken.

## Introduction

Obesity is a condition in which the natural energy reserve, stored in the fatty tissue of humans and other mammals, is increased to a point where it is associated with certain health conditions or increased mortality [1].

Obesity is a major public health problem in developed countries especially in the United States, with one-third to one-half of adults affected. Nowadays, it also occurs in the developing countries. Obesity is associated with five out of ten leading causes of death and disability such as heart disease, diabetes, cancer, hypertension and stroke. An estimated 300,000 people die each year of illnesses related to obesity, more than the number killed by pneumonia, motor vehicle accidents and airlines crashes combined [2]. Since 1991, the percentage of obese Americans has increased by 74%. More than 21 million U.S men and over 23 million women are obese [3].

The most comprehensive data on the prevalence of obesity worldwide are those of the WHO MONICA project. The main conclusion drawn from the project was that obesity prevalence is increasing worldwide at an alarming rate in both developed and developing countries. In many developing countries, obesity coexists with undernutrition. Although still relatively uncommon in African and Asian countries, obesity is more prevalent in urban than rural populations. In economically advanced regions, prevalence rates may be as high as in developed countries. Another significant finding from the WHO MONICA project is that women generally have higher rates of obesity than men [4].

Many other studies have also shown that the prevalence of obesity among women was higher than men. The age range of 25–44 years is the time when women tend to gain the greatest amount of weight. Among women of childbearing age, one potential pathway for the development of obesity has been through the retention of gestational weight gain [5].

For the past two decades, rapid and marked socioeconomic advancement in Malaysia has brought about significant changes in the lifestyles of communities. These include significant changes in the dietary patterns of Malaysians. Changes in meal patterns are also evident where more families eat out, busy executives skip meals, and the younger generations miss breakfast and rely too much on fast food. In addition, communities have become generally more sedentary. Woman have more frequent opportunities to consume food and are more likely to have greater volumes of food available because they traditionally prepare meals for their families [6]. However, more women are eating outside their homes nowadays, as well as buying home food from restaurants, food-stalls and fast-food centers for their families.

Many Malaysians are at huge health risk because they are overweight or obese. The National Health and Morbidity Survey 2, conducted by the Ministry of Health in 1996 and 1997, found that 4.4 per cent and 16.6 per cent of the population were obese and overweight respectively. Based on adult population between the ages of 20 and 59 years old, that translates to about 450,000 obese and 1.72 million overweight adult Malaysians. Using the World Health Organization (WHO) guidelines of Body Mass Index (BMI) ≥ 25.0 for overweight and BMI ≥ 30.0 for obesity, it was reported that in Malaysian adult males, 15.1% were overweight and 2.9% obese while in adult females, 17.9% were overweight and 5.7% obese [7].

The WHO MONICA project found that good -quality and nationally representative data for countries in South East Asia were unavailable [8]. However, two studies from Thailand found that diet-related chronic diseases, including obesity are increasing in affluent urban populations and obesity is significantly higher among women as compared to men. As many countries in South East Asia, including Malaysia are currently going through the "nutrition transition" (change in structure of diet, reduced physical activity and rapid increases in the prevalence of obesity), the WHO MONICA project emphasizes on the special need to collect good-quality, nationally representative obesity prevalence data [4].

Therefore, the aim of this study was to determine the prevalence and associated factors of obesity among adult women in Selangor, Malaysia. The findings of this study can provide some baseline data on the magnitude of this problem, with emphasis on women in Selangor, as well as identify factors to focus on when addressing the problem of obesity among women.

## Method

Selangor is one of the eleven states in Peninsular Malaysia. With an area of approximately 8,000 sq. km, Selangor extends along the west coast of Peninsular Malaysia at the northern outlet of the Straits of Malacca. It is one of the most prosperous states in Malaysia, with a population of about 3.75 million inhabitants.

This community based cross sectional study was conducted in Selangor in January 2004. All districts were included. Multi stage stratified proportionate to size sampling method was used to select households in each district. No distinction was made between urban, semi-urban or rural areas. Women aged 20–59 years old were included in this study and contacted via home visits.

Exclusion criteria included foreigners and known psychiatric cases. A standardized pre-tested structured questionnaire was used. Height and weight measurements were taken from the respondents by a trained Research Assistant using calibrated equipments (Seca body metre for height and tanita measuring scale for weight).

The questionnaire consisted of 4 parts which consisted of questions on socio- demographic (age, ethnicity, religion, education level, occupation, monthly income, marital status), Obstetric & Gynaecology history, body mass index (BMI), and the Patient Health Questionnaire (PHQ-9) which was used to determine the presence or absence of depressive symptoms.

The WHO criteria for obesity based on the BMI guidelines was used in this study. BMI equals weight in kilograms divided by height in metres squared (BMI = kg/m^2^). Using BMI, it is possible to classify the degree of obesity by reference to internationally accepted ranges, commencing from underweight (BMI < 18.5 kg/m^2^), normal (BMI 18.5–24.9 kg/m^2^), overweight (BMI 25.0–29.9 kg/m^2^) and obese (BMI ≥ 30 kg/m^2^) [9].

The Patient Health Questionnaire (PHQ-9) was developed by Drs. Robert L Spitzer, Janet BW Williams, Kurt Kroenke and colleagues. It was developed from the Primary Care Evaluation of Mental Disorders Patient Health Questionnaire (PRIME-MD PHQ) which was designed to facilitate the recognition and diagnosis of the most common mental disorders. It is a self-report questionnaire and consists of 9 questions that identify depressive symptoms. The PHQ Depression Severity Index score is used to calculate for the presence of depressive symptoms [10].

The questionnaire was translated and validated in Bahasa Malaysia. Pre-testing was done in another location not included in the study. Data was analyzed using the computer program "Statistical Package for the Social Sciences" (SPSS) version 11.5. Descriptive statistics were used for all the variables studied. Pearson Chi-square, Odds ratio and 95% Confidence Interval were used to test the association and risk between each factor and depressive symptoms.

## Results

Out of 1032 women, 972 agreed to participate in this study, giving a response rate of 94.19%. Age of the respondents ranged from 20–59 years old. The mean age was 37.91 ± 10.91 with 95% CI = 37.2–38.6. The prevalence of obesity among respondents was 16.7% (mean = 1.83 ± 0.373).

Table 1 shows the socio-demographic profile of the respondents. Majority of the respondents were aged between 20 to 49 years old (82.2%). About half of them were Malays (54.9%), followed by Indians (23.4%) and Chinese (20.0%). Majority were Muslims (56.3%), followed by Hindus (21.8%) and Buddhist (17.0%). Most of the respondents attended school (94.0%) and had formal education (94.1%). However, only 40% of them were working and majority of their income was less than RM 500 per month (67.9%). Most of the respondents were married (76.7%).

**Table 1:** Socio-demographic profile of the respondents (n = 972)

**Profile of the respondents**	**n**	**%**
**Age**		
		
20–49 years	799	82.2
50–59 years	173	17.8
		
**Race**		
		
Malay	534	54.9
Chinese	194	20.0
Indian	227	23.4
Others	17	1.7
		
**Religion**		
		
		
Muslim	547	56.3
Buddhist	165	17.0
Christian	44	4.5
Hindu	212	21.8
Others	4	0.4
		
**School**		
		
Yes	914	94.0
No	58	6.0
		
**Education level**		
		
Formal education	915	94.1
No formal education	57	5.9
		
**Occupation**		
		
Yes	389	40.0
No	583	60.0
		
**Monthly salary**		
		
^++ ^< RM 500	660	67.9
^++ ^≥RM 500	312	32.1
		
**Marital Status**		
		
Yes	815	83.8
No	157	16.2

* p < 0.05 = significant

^++ ^RM = Malaysian Dollars

Among the respondents who were married (n = 746), 2.9% suffered from miscarriage within the last 6 months, 5.9% had difficulty in getting pregnant for the past 2 years and 6.3% had given birth within the last 6 months.

Table 2 shows the association between BMI and socio demographic profile of the respondents. There was significant association between obesity and age (p = 0.013, OR = 0.65, 95% CI = 0.47–0.91), ethnicity (p = 0.001), religion (p = 0.002), school attendance (p = 0.020, OR = 0.57, 95% CI = 0.36–0.89), education level (p = 0.016, OR = 0.55, 95% CI = 0.35–0.87) and marital status (p = 0.001, OR = 2.63, 95% CI = 1.90–3.65) but there was no significant association with occupation and total family income of the respondents.

**Table 2:** Association of socio-demographic factors and body mass index (BMI) among the respondents (n = 891)

**Profile of the respondents**	**Obese BMI ≥ 30 n(%)**	**Non-Obese BMI < 30 n(%)**	**p value**	**OR**	**95% CI**
**Age**					
					
20–49 years	112(15.3)	621(84.7)	0.013*	0.65	0.47–0.91
50–59 years	37(23.4)	121(76.6)			
					
**Race**					
					
Malay	95(19.4)	394(80.6)	0.001*		
Chinese	11(6.2)	166 (93.8)			
Indian	40(19.0)	170(81.0)			
Others	3(20.0)	12(80.0)			
					
**Religion**					
					
Muslim	97(19.4)	404(80.6)	0.002*		
Buddhist	8(5.3)	142(94.7)			
Christian	7(17.5)	33(82.5)			
Hindu	36(18.3)	161(81.7)			
Others	1(33.3)	2(61.7)			
					
**School**					
					
Yes	134(16.0)	704(84.0)	0.020*	0.57	0.36–0.89
No	15(28.3)	38(71.7)			
					
**Education level**					
					
Formal education	134(16.0)	705(84.0)	0.016*	0.55	0.35–0.87
No formal education	15(28.8)	37(71.2)			
					
**Occupation**					
					
Yes	54(15.1)	303(84.9)	0.296	0.85	0.63–1.16
No	95(17.8)	439(82.2)			
					
**Total family income**					
					
< RM 500	108(17.9)	497(82.1)	0.189	1.25	0.89–1.73
≥ RM 500	41(14.3)	245(85.7)			
					
**Marital Status**					
					
Yes	139(18.6)	607(81.4)	0.001*	2.70	1.50–5.01
No	10(6.9)	135(93.1)			

* p < 0.05 = significant

Table 2 also shows that respondents from the age group 50–59 years old had higher prevalence of obesity (58.2%) compared to respondents of the 20–49 years old age group (45.6%). There was significant association between obesity and race in this study where the prevalence of obesity was highest among the other races (20.0%).

Further analyses revealed that there was significant association of obesity between Malay and Chinese (p = 0.000, OR = 3.13, 95% CI = 1.72–5.70), Indian and Chinese (p = 0.000, OR = 0.33, 95% CI = 0.17–0.62), and other races and Chinese (p = 0.049, OR = 0.09, 95% CI = 0.99). There was also significant association between obesity and religion in this study, where further analyses found that obesity was significantly associated between Islam and Buddhist (p = 0.000, OR = 3.63 95% CI = 1.81–7.30), Christians and Buddhist (p = 0.011, OR = 0.31 95% CI = 0.12–0.79), Hindu and Buddhist (p = 0.000, OR = 0.30 95% CI = 0.14–0.61) and other religions and Buddhist (p = 0.041, OR = 0.16 95% CI = 0.03–0.91).

Analysis for association in Table 3 and Table 4 was done for respondents who only had a BMI measurement (n = 891). There was no significant association between obesity and depressive symptoms (Table 3). Table 4 shows the association between obesity and miscarriage within the last 6 months; difficulty in getting pregnant for the past 2 years and giving birth within the last 6 months. There was a significant association between respondents who suffered a miscarriage within the last 6 months and obesity (p = 0.023).

**Table 3:** Association between Obesity with Depressive Symptoms (n = 891)

**Profile of the respondents**	**Depressive n(%)**	**Not Depressive n(%)**	**p value**	**OR**	**95% CI**
**Body Mass Index**					
					
**Obese**					
(BMI ≥ 30)	15(10.1)	134(89.9)	0.427	1.25	0.73–2.13
					
**Non-Obese**					
(BMI < 30)	60(8.1)	682(91.9)			

**Table 4:** Association between Obesity and history of having a miscarriage within last 6 months, difficulty getting pregnant during past 2 years and giving birth within last 6 months (n = 746)

**Profile of the respondents**	**Obese BMI ≥ 30 n(%)**	**Non-Obese BMI < 30 n(%)**	**p value**	**OR**	**95% CI**
**Had a miscarriage within the last 6 months**					
					
Yes	0(0.0)	22(100.0)	0.023*	1.24	1.19–1.28
No	139(19.2)	585(80.8)			
					
**Having difficulty getting pregnant past 2 years**					
					
Yes	6(13.6)	38(86.4)	0.380	0.72	0.34–1.54
No	133(18.9)	569(81.1)			
					
**Have you given birth within the last 6 months**					
Yes	7(14.9)	40(85.1)	0.496	0.79	0.39–1.59
No	132(18.9)	567(81.1)			

* p < 0.05 = significant

## Discussion

The prevalence of obesity is increasing rapidly in both developed and developing countries. It has reached epidemic proportions globally, and evidence suggests that the situation is likely to get worse especially among women. One of the reasons for this is because women tend to gain greatest amount of weight during their childbearing age (between 25–44 years old) [5].

This study found that age was significantly associated with obesity (p = 0.013). Prevalence of obesity was higher among respondents with increasing age. The National Health and Morbidity Survey 2, conducted by the Malaysian Ministry of Health in 1996 and 1997, also found that BMI increases with age. Decrease in height as a person ages has been quoted as one of the reasons BMI increases with age [9].

Results in this study showed that other races such as Orang Asli, Eurasians and Sikhs had the highest prevalence of obesity followed by Malays, Indians and Chinese. Further analysis revealed that there was significant association of obesity between Malay and Chinese (p = 0.000, OR = 3.13, 95% CI = 1.72–5.70), Indian and Chinese (p = 0.000, OR = 0.33, 95% CI = 0.17–0.62), and other races and Chinese (p = 0.049, OR = 0.09, 95% CI = 0.99). There was also significant association between obesity and religion (p = 0.002), where other religions had the highest prevalence of obesity followed by Muslims, Hindus, Christians and Buddhist. This finding is supported by Malaysia's National Health and Morbidity Survey 2 (1996 – 1997) which found that obesity was significantly associated with ethnicity. However, their findings showed that Indians had the highest prevalence of obesity followed by Malays, other indigenous and lastly Chinese [9].

In their study among whites, blacks and Hispanics, Paeratakul et al (2002) also found that ethnicity was significantly associated with body mass index, where the prevalence of overweight and obesity was found to be higher in the ethnic minority population especially in black women compared to whites [11].

There was significant association between obesity with school attendance (p = 0.020) and educational level (p = 0.016) in this study. Prevalence of the obesity was significantly higher among respondents who had not attended school (28.3%) compared to respondents who had attended school (16.0%). Respondents with no formal education also had a higher prevalence of obesity (28.8%) compared to those with formal education (16.0%). This finding is similar to the study done by Parkes (2003), which found that respondents with no schooling and no formal education had significantly higher BMI than those with qualifications [12].

This study showed that marital status was significantly associated with obesity (p = 0.001), where respondents who were married had a higher prevalence of obesity (18.6%) compared to those who were still unmarried (6.9%). This finding is supported by Jeffery (2002) who found that marriage was associated with a significant 2-year weight gain and divorce with a significant 2-years weight loss. The effects of marriage and divorce on weight may be due to the influence of marriage on inducement to eat (e.g., shared meals) or on motivation for weight control [13].

This study also found that there was significant association between obesity and respondents who suffered a miscarriage within the last 6 months (p = 0.023), where respondents who did not suffer a miscarriage had a higher prevalence of obesity (19.2%) compared to respondents who suffered a miscarriage (0.0%). The result from this study differs from the study done by Lanshen et al (2004) among obese women in U.K. which found that obesity is associated with increased risk of miscarriage [14].

## Conclusion

The results of this study found that socio-demographic factors such as age, ethnicity, religion, schooling, education level and marital status were significantly associated with obesity among women aged 20–59 years old. As for obstetrics and gynaecology factors, only the history of suffering a miscarriage within the past 6 months was significantly associated with obesity in this study.

The findings of this study can provide baseline data for monitoring the effectiveness of national programs for the prevention and control of obesity in Malaysia, especially among women. These programs can focus on the factors found to be significantly associated with obesity among women in their reproductive years to ensure maximum benefit while focusing on this group of women. Resources for the prevention and control of obesity can be mobilized and allocated based on the factors identified to be associated with obesity. Further studies need to be done to assess the main contributing factors associated with obesity in this group of women.
